# Effects of Brimonidine, Latanoprost, and Omidenepag on Tunicamycin-Induced Endoplasmic Reticulum Stress and Fibrosis in Human Trabecular Meshwork Cells

**DOI:** 10.3390/biom15030389

**Published:** 2025-03-08

**Authors:** Mengxuan Liu, Megumi Honjo, Reiko Yamagishi, Makoto Aihara

**Affiliations:** 1 Department of Ophthalmology, Graduate School of Medicine, The University of Tokyo, Tokyo 113-8655, Japan; 2 Department of Ophthalmology, Ruijin Hospital Affiliated Medical School, Shanghai Jiaotong University, Shanghai 200025, China

**Keywords:** human trabecular meshwork cells, tunicamycin, endoplasmic reticulum stress, fibrosis, brimonidine, latanoprost, omidenepag

## Abstract

This study evaluated the effects of α2-adrenergic agonist, prostaglandin F2α analog, and EP2 receptor agonist on tunicamycin-induced endoplasmic reticulum (ER) stress and fibrosis in human trabecular meshwork (TM) cells. Human TM cells were treated with tunicamycin for 24 h, followed by cotreatment with brimonidine (BRI), latanoprost (LAT), or omidenepag (OMD). Immunocytochemistry was used to assess expressions of collagen type I alpha 1 chain (COL1A1), fibronectin, F-actin, and alpha-smooth muscle actin (α-SMA). Western blotting was performed to evaluate levels of C/EBP homologous protein (CHOP), 78-kDa glucose-regulated protein (GRP78), and splicing X-box binding protein-1 (sXBP-1). Real-time qPCR was used to examine the mRNA expressions of COL1A1, connective tissue growth factor (CTGF), fibronectin, α-SMA, CHOP, GRP78, and sXBP-1. Expressions of COL1A1, CTGF, F-actin, fibronectin, α-SMA, CHOP, GRP78, and sXBP-1 significantly increased after tunicamycin treatment. BRI cotreatment significantly downregulated the mRNA and protein expressions of GRP78, and LAT or OMD cotreatment significantly reduced the CHOP and sXBP-1 expressions compared to the tunicamycin-treated group. BRI, LAT, or OMD cotreatment significantly attenuated cellular cytoskeletal changes and the increase of fibrosis markers such as COL1A1, CTGF, fibronectin, and α-SMA. In addition, COL1A1 mRNA expression was significantly lowered with LAT or OMD cotreatment compared to the BRI-cotreated group. Cotreatment with α2-adrenergic agonist, prostaglandin F2α analog, or EP2 receptor agonist alleviates tunicamycin-induced ER stress in human TM cells.

## 1. Introduction

Primary open-angle glaucoma (POAG), the most common form of glaucoma, is characterized by elevated intraocular pressure (IOP), a major risk factor that leads to irreversible vision loss [1,2,3]. The trabecular meshwork (TM) constitutes the conventional aqueous humor (AH) outflow pathway which plays a crucial role in AH regulation and IOP maintenance [4,5]. The ciliary body continuously secretes AH, which drains from the anterior chamber through the TM, juxtacanalicular connective tissue, and Schlemm’s canal tissue. An excessive accumulation of fibrosis and extracellular matrix materials in the TM increases AH outflow resistance, contributing to IOP elevation in POAG patients [6,7].

A significant decrease in the number of TM cells and associated dysfunctions are key pathological changes in POAG [8,9]. The precise mechanisms responsible for TM cell number decline remain unclear; however, apoptosis is suggested to be one of the most important causes, which is triggered by endoplasmic reticulum (ER) stress [10]. The ER is responsible for the synthesis, folding, and maturation of secretory and membrane proteins. The accumulation of unfolded and misfolded proteins in the ER results in a stressful condition known as ER stress under various disturbances [11,12]. The unfolded protein response (UPR) is activated through transcriptional mechanisms to restore ER function and maintain cellular homeostasis by the activation of ER chaperones 78-kDa glucose-regulated protein (GRP78), splicing X-box binding protein-1 (sXBP-1), and protein-folding enzymes [11,12,13,14,15]. When the chronic ER stress persists and the UPR adaptive response fails to resolve the overwhelmed unfolded and misfolded proteins, the UPR can trigger apoptotic cell death through the induction of C/EBP homologous protein (CHOP) [11,12,13]. In addition, excessive and sustained ER stress is associated with TM dysfunction, leading to IOP elevation and POAG development [13,16].

Treatments for POAG focus primarily on reducing IOP through medications, surgical treatments, or laser therapy [17]. Of these, medications are the first-line treatment for most glaucoma patients [18]. Latanoprost (LAT) is a widely used prostaglandin F2α analog with strong efficacy, approved as the first-line drug for reducing IOP in glaucoma patients [19,20]. Brimonidine (BRI), a highly selective α2-adrenergic agonist, lowers IOP by reducing AH production and increasing uveoscleral outflow and has been marketed since 2012 [21]. Omidenepag (OMD) isopropyl is an EP2 receptor agonist with a non-prostaglandin structure, which enhances both trabecular outflow and uveoscleral outflow with a reduced number of adverse effects, and was approved in 2018 [22,23,24]. Reports found that an imbalance of Ca^2+^ causes ER stress, and that the α2-adrenergic agonist, prostaglandin F2α analog, and EP2 receptor agonist play an important role in the Ca^2+^ regulation [25,26,27,28,29]. Accordingly, we chose these medications to evaluate and compare the effects on ER stress-mediated human TM cells in vitro in the present study.

A further understanding of the clinical medications underlying TM dysfunction may potentially promote the clinical therapy toward more efficient treatments for POAG patients. However, these medications have not been directly investigated in human TM cells under ER stress in vitro. Tunicamycin is a kind of protein glycosylation inhibitor, which is used as an inducer of ER stress in cells [11,14,30]. Therefore, we investigated and compared the effects of BRI, LAT, and OMD on tunicamycin-induced ER stress in human TM cells in vitro.

## 2. Materials and Methods

### 2.1. Cell Culture

Primary human TM cells were isolated from donor eyes without glaucoma (46, 52, and 55 years old) and characterized as described previously [31,32], in accordance with the method of Keller et al. [33]. Ethical approval was obtained from the institutional review board of the University of Tokyo and registered with the University Hospital Medical Information Network Clinical Trials Registry of Japan (ID: UMIN000027137). All procedures conformed to the tenets of the Declaration of Helsinki. The cells were cultured in Dulbecco’s Modified Eagle Medium supplemented with 10% fetal bovine serum and antibiotic-antimycotic solution (100×) (Sigma-Aldrich, St. Louis, MO, USA) at 37 °C with 5% CO_2_. Passages of human TM cells between three and six were used in the present study. To assess the effects of ER stress on human TM cells, confluent cells were incubated in a serum-free medium for 24 h before treatment. The cells were treated with various concentrations of tunicamycin (Wako Pure Chemical Industries, Ltd., Osaka, Japan), with or without BRI (Wako Pure Chemical Industries, Ltd., Osaka, Japan) at 1 or 10 μM; LAT (Cayman Chemical Company, Ann Arbor, MI, USA) at 0.1, 1, or 10 μM; and OMD (Santen Pharmaceutical Co., Ltd., Osaka, Japan) at 0.1, 1, or 10 μM. The concentrations of BRI and LAT were set to their reported concentrations in the anterior chamber, and tissue distribution data following OMD isopropyl (EYBELIS™; Santen Pharmaceutical) in the eyes of monkeys were used to estimate the OMD concentration in the TM at 59.8 nM [34,35,36]. In the control group, the medium was changed at the same points without adding tunicamycin or medications. All experiments were performed at least three times, and the consistency of the experiment was confirmed using biological triplicates.

### 2.2. WST-1 Assay

Cellular viability of human TM cells following tunicamycin treatment was evaluated using the WST-1 assay (Dojindo Co., Tokyo, Japan) according to the manufacturer’s protocol. Briefly, human TM cells were cultured overnight in 96-well plates before different concentrations (0, 0.001, 0.01, 0.1, and 1 μg/mL) of tunicamycin treatment. Then, cellular viability was measured using a multimode plate reader (PerkinElmer, Waltham, MA, USA).

### 2.3. Immunocytochemistry

Immunocytochemistry was performed as previously described [31,37]. The primary antibodies were anti-collagen type I alpha 1 chain (COL1A1, 1:400; Rockland Immunochemicals, Limerick, PA, USA), anti-alpha-smooth muscle actin (α-SMA, 1:200; Dako, Agilent, Santa Clara, CA, USA), anti-fibronectin (1:400; Santa Cruz Biotechnology, Dallas, TX, USA), and rhodamine phalloidin (7:1000; Thermo Fisher Scientific, Waltham, MA, USA). Alexa Fluor 488- and Alexa Fluor 594-conjugated secondary antibodies (1:1000) were used and purchased from Thermo Fisher Scientific (Waltham, MA, USA). Images were obtained using a BX51 fluorescence microscope (Olympus, Tokyo, Japan), and quantitative analysis of the immunocytochemistry was conducted using ImageJ software (ver. 1.49, National Institutes of Health, Bethesda, MD, USA).

### 2.4. Real-Time Quantitative Polymerase Chain Reaction (qPCR)

The human TM cells were lysed using ISOGEN (Nippon Gene, Tokyo, Japan), and total mRNA was isolated with chloroform and isopropyl alcohol. cDNA was synthesized from the isolated mRNA using a PrimeScript RT Reagent Kit (Takara Bio, Shiga, Japan). The mRNA levels were quantified using the ΔΔCt method, as previously described [38]. Primer sequences used in this study were derived from previously published reports and purchased from the Hokkaido System Science (Hokkaido, Japan). The PCR primers used were as follows: CHOP, forward, 5′-AGAACCAGGAAACGGAAACAGA-3′ and reverse, 5′-TCTCCTTCATGCGCTGCTTT-3′; COL1A1, forward, 5′-CAGCCGCTTCACCTACAGC-3′ and reverse, 5′-TTTTGTATTCAATCACTGTCTTGCC-3′; connective tissue growth factor (CTGF), forward, 5′-CTCCTGCAGGCTAGAGAAGC-3′ and reverse, 5′-GATGCACTTTTTGCCCTTCTT-3′; fibronectin, forward, 5′-AAACCAATTCTTGGAGCAGG-3′ and reverse, 5′-CCATAAAGGGCAACCAAGAG-3′; GRP78, forward, 5′- CATCACGCCGCTCTATGTCG-3′ and reverse, 5′-CGTCAAAGACCGTGTTCTTCTCG-3′; sXBP-1, forward, 5′-CTGAGTCCGAATCAGGTGCAG-3′ and reverse, 5′-ATCCATGGGGAGATGTTCTGG-3′; α-SMA, forward, 5′-CCGACCGAATGCAGAAGGA-3′ and reverse, 5′-ACAGAGTATTTGCGCTCCGAA-3′; and GAPDH, forward, 5′-AATTCCATGGCACCGTCAAG-3′ and reverse, 5′-ATCGCCCCACTTGATTTTGG-3′. Gene expression levels were normalized relative to GAPDH.

### 2.5. Western Blotting

Western blotting was performed as described previously [31]. Briefly, cell lysates were collected in RIPA buffer (Thermo Fisher Scientific) containing protease inhibitors (Roche Diagnostics, Basel, Switzerland) after treatments. Protein concentrations were measured using the BCA Protein Assay Kit (Thermo Fisher Scientific), with bovine serum albumin as a standard. Protein extracts were separated by SDS-PAGE and transferred to PVDF membranes (Bio-Rad Laboratories, Hercules, CA, USA). Subsequently, the membranes were immersed in primary antibodies overnight at 4 °C. The primary antibodies were anti-CHOP (1:1000; Cell Signaling Technology, Inc., Danvers, MA, USA), anti-GRP78 (1:1000; Santa Cruz Biotechnology, Dallas, TX, USA), anti-sXBP-1 (1:1000; Cell Signaling Technology, Inc.), and anti-β-tubulin (1:1000; Wako Pure Chemical Industries, Ltd.). After washing, membranes were incubated with horseradish peroxidase-conjugated anti-mouse or anti-rabbit secondary antibodies (1:2000–1:5000; Thermo Fisher Scientific) for 1 h at room temperature. The membranes were reacted with ECL substrate (Thermo Fisher Scientific) followed by ImageQuant LAS 4000 mini (GE Healthcare, Chicago, IL, USA). The bands were quantified using ImageJ software.

### 2.6. Statistical Analysis

Statistical analyses were performed using SPSS version 22.0 (IBM Corp., Armonk, NY, USA). The results are presented as mean ± standard deviation (SD). Differences between groups were compared with a one-way ANOVA followed by Tukey’s post-hoc test. *p* < 0.05 was considered as statistically significant.

## 3. Results

### 3.1. Effects of Tunicamycin on Human TM Cell Viability

We treated cultured human TM cells with different concentrations (0, 0.001, 0.01, 0.1, and 1 μg/mL) of tunicamycin to evaluate the effects of tunicamycin on cellular viability in human TM cells (Supplementary Figure S1). After treatment with different concentrations of tunicamycin for 24 h, the results indicated that it did not induce significant changes with these concentrations in human TM cell viability compared to the control. With a high concentration of 10, 30, and 50 μg/mL tunicamycin treatment, it significantly reduced human TM cell viability compared to the control (Supplementary Figure S2).

### 3.2. Effects of Tunicamycin on the mRNA Expressions of CHOP, GRP78, and sXBP-1 in Human TM Cells

**Figure 1:**
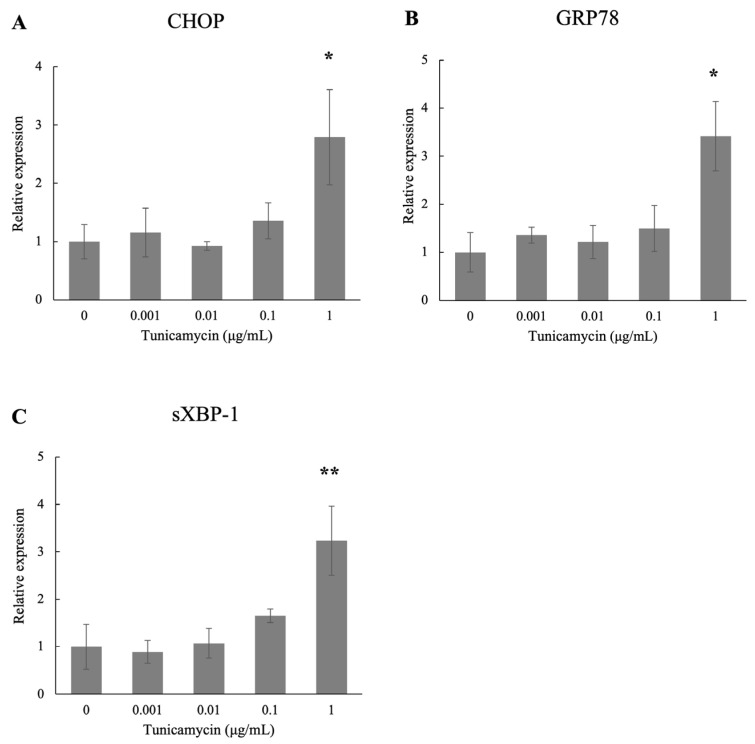
Real-time qPCR analysis of (**A**) CHOP, (**B**) GRP78, and (**C**) sXBP-1 mRNA expressions after treatment with different concentrations (0, 0.001, 0.01, 0.1, and 1 μg/mL) of tunicamycin in human TM cells in vitro. The results showed that 1 μg/mL tunicamycin treatment significantly increased the mRNA levels of these markers compared to the control. GAPDH was used as an internal control for normalization. Data are presented as the mean ± standard deviation. * *p* < 0.05, ** *p* < 0.01.

We investigated the effects of tunicamycin on the mRNA expressions of the ER stress markers CHOP, GRP78, and sXBP-1 in human TM cells with real-time qPCR in vitro (Figure 1). Treatment with 1 μg/mL tunicamycin significantly upregulated the expressions of these markers compared to the control and 0.1 μg/mL tunicamycin treatment.

### 3.3. Effects of Different BRI, LAT, and OMD Concentrations on CHOP, GRP78, and sXBP-1 mRNA Expressions in Tunicamycin-Treated Human TM Cells

**Figure 2:**
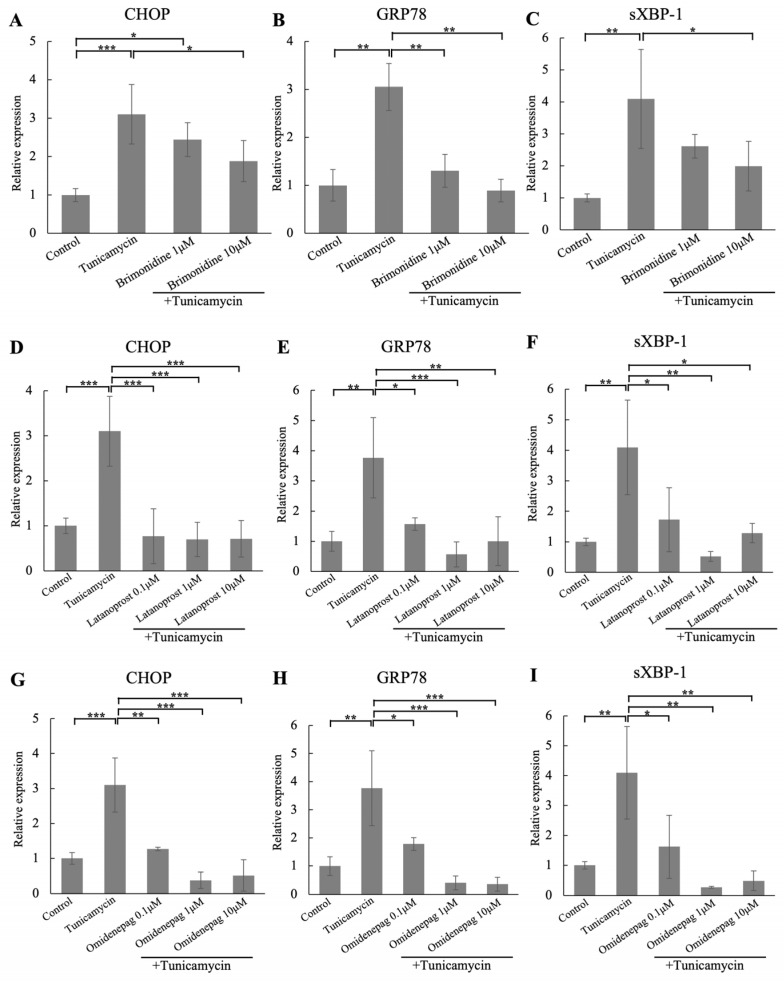
Real-time qPCR analysis of (**A**,**D**,**G**) CHOP, (**B**,**E**,**H**) GRP78, and (**C**,**F**,**I**) sXBP-1 mRNA expressions after treating with different concentrations of BRI, LAT, OMD, with or without tunicamycin, in human TM cells. The relative mRNA expressions of CHOP, GRP78, and sXBP-1 were significantly elevated after tunicamycin treatment. Cotreatment with BRI, LAT or OMD significantly decreased the mRNA expressions of these ER stress markers compared to the tunicamycin-treated group. GAPDH was used as an internal control for normalization. Data are presented as the mean ± standard deviation. * *p* < 0.05, ** *p* < 0.01, *** *p* < 0.001.

We evaluated different BRI, LAT, and OMD concentrations on the ER stress markers of CHOP, GRP78, and sXBP-1 mRNA expressions in tunicamycin-treated human TM cells (Figure 2). Tunicamycin treatment significantly increased these ER stress markers compared to the control. Cotreatment with 1 μM BRI significantly reduced GRP78 mRNA expression, and 10 μM BRI cotreatment significantly decreased the CHOP, GRP78, and sXBP-1 mRNA expressions compared to the tunicamycin-treated group, although there was no significant difference between 1 and 10 μM BRI. Also, cotreatment with various concentrations of LAT (0.1, 1, and 10 μM) or OMD (0.1, 1, and 10 μM) significantly reduced the upregulated mRNA expressions of CHOP, GRP78, and sXBP-1. As no significant differences were observed between various concentrations of BRI, LAT, and OMD, subsequent experiments were conducted using concentrations with reported anterior chamber concentrations or tissue distribution for these drugs. The BRI physiological concentration in aqueous humor was reported to be around 1.5 μM, and LAT in aqueous humor around 0.5 μM [35,36]. Tissue distribution data from Santen Pharmaceutical in the eyes of monkeys were used to estimate the OMD concentration in the TM that reported at 0.598 μM. Accordingly, the concentrations were set up based on their physiological concentrations of BRI with 1 and 10 μM, LAT and OMD with 0.1, 1, and 10 μM in this study to compare the effectiveness of these three drugs.

### 3.4. Comparison of BRI, LAT, and OMD on CHOP, GRP78, and sXBP-1 mRNA Amount in Tunicamycin-Treated Human TM Cells

**Figure 3:**
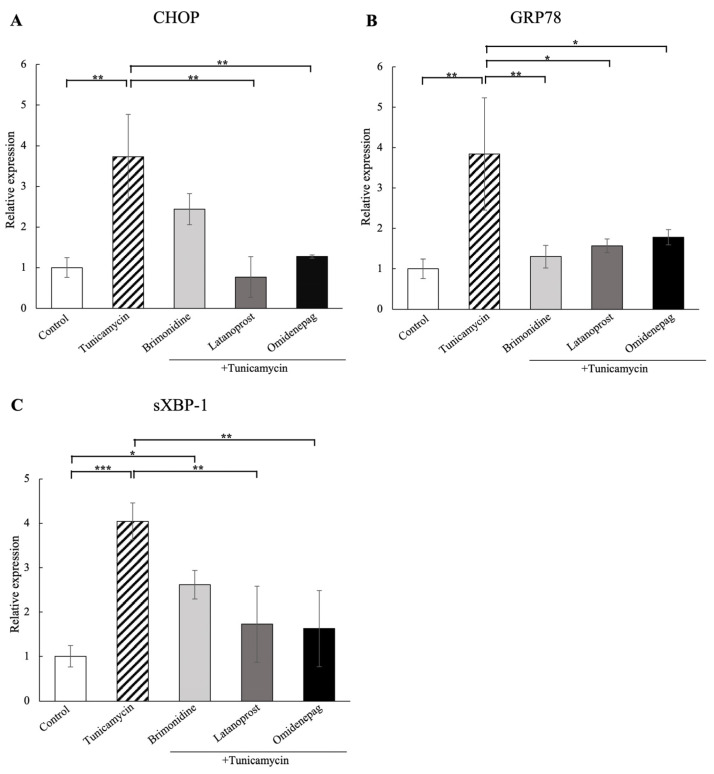
Real-time qPCR analysis of (**A**) CHOP, (**B**) GRP78, and (**C**) sXBP-1 mRNA expressions in human TM cells treated with 1 μM BRI, 0.1 μM LAT, and 0.1 μM OMD, with or without tunicamycin. Tunicamycin treatment significantly upregulated the relative mRNA expressions of CHOP, GRP78, and sXBP-1. Cotreatment with BRI significantly downregulated GRP78 mRNA expression, and LAT or OMD cotreatment significantly reduced the mRNA expressions of CHOP, GRP78, and sXBP-1 compared to the tunicamycin-treated group. GAPDH was used as an internal control for normalization. Data are presented as the mean ± standard deviation. * *p* < 0.05, ** *p* < 0.01, *** *p* < 0.001.

We evaluated the effects of 1 μM BRI, 0.1 μM LAT, and 0.1 μM OMD on the mRNA expressions of the ER stress markers CHOP, GRP78, and sXBP-1 in human TM cells treated with tunicamycin (Figure 3). Tunicamycin treatment significantly increased the relative mRNA expressions of these ER stress markers compared to the control. Additional treatment with BRI significantly reduced GRP78 mRNA expression, and cotreatment with LAT or OMD significantly downregulated the mRNA expressions of CHOP, GRP78, and sXBP-1 compared to the tunicamycin-treated group.

### 3.5. Effects of BRI, LAT, and OMD on CHOP, GRP78, and sXBP-1 Protein Expressions in Tunicamycin-Treated Human TM Cells

**Figure 4:**
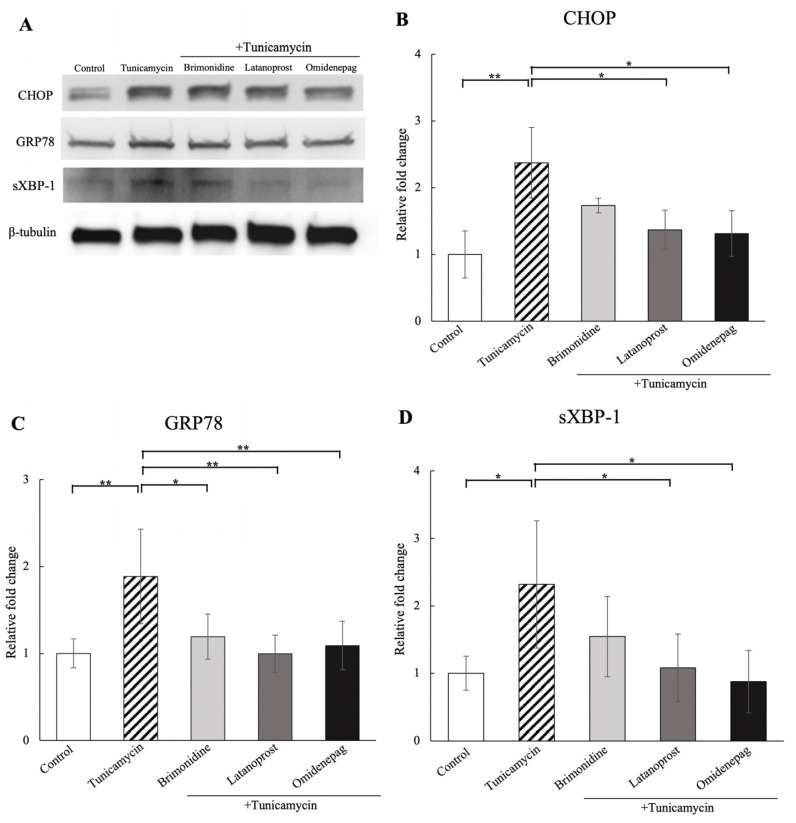
Western blotting analysis of tunicamycin on ER stress markers after treatment of 1 μM BRI, 0.1 μM LAT, 0.1 μM OMD, with or without tunicamycin, in human TM cells. Representative bands are shown in (**A**). The protein amounts of (**B**) CHOP, (**C**) GRP78, and (**D**) sXBP-1 increased significantly after tunicamycin treatment. BRI cotreatment significantly lowered the GRP78 protein amount, and LAT or OMD cotreatment significantly decreased the protein levels of CHOP, GRP78, and sXBP-1 compared to the tunicamycin-treated group. Results were expressed relative to the loading control (β-tubulin). Data are presented as mean ± standard deviation. * *p* < 0.05, ** *p* < 0.01. Original western blots can be found at Supplementary Materials.

With Western blotting analysis, the effects of 1 μM BRI, 0.1 μM LAT, and 0.1 μM OMD on ER stress protein markers of CHOP, GRP78, and sXBP-1 were examined in tunicamycin-treated human TM cells in vitro (Figure 4). The results showed that tunicamycin treatment significantly upregulated the protein expressions of these ER stress markers compared to the control. Additional treatment with BRI significantly decreased GRP78 protein expression, and cotreatment with LAT or OMD significantly reduced the protein expressions of CHOP, GRP78, and sXBP-1 compared to the tunicamycin-treated group.

### 3.6. Effects of Different BRI, LAT, and OMD Concentrations on COL1A1, CTGF, Fibronectin, and α-SMA mRNA Expressions in Tunicamycin-Treated Human TM Cells

**Figure 5:**
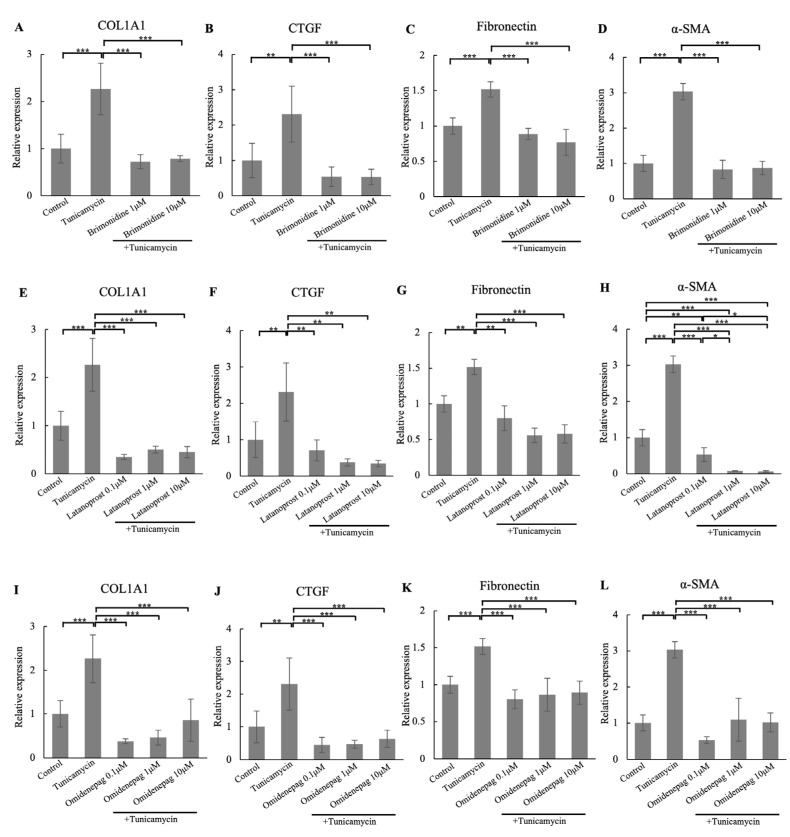
Real-time qPCR analysis of (**A**,**E**,**I**) COL1A1, (**B**,**F**,**J**) CTGF, (**C**,**G**,**K**) fibronectin, and (**D**,**H**,**L**) α-SMA mRNA expressions after treating with different concentrations of BRI, LAT, OMD, with or without tunicamycin, in human TM cells. The relative mRNA expressions of COL1A1, CTGF, fibronectin, and α-SMA were significantly upregulated after tunicamycin treatment. With BRI, LAT, or OMD, cotreatment significantly downregulated the mRNA expressions of these fibrosis markers compared to the tunicamycin-treated group. GAPDH was used as an internal control for normalization. Data are presented as the mean ± standard deviation. * *p* < 0.05, ** *p* < 0.01, *** *p* < 0.001.

We investigated the effects of different concentrations of BRI, LAT, and OMD on the mRNA expressions of fibrosis markers COL1A1, CTGF, fibronectin, and α-SMA in tunicamycin-treated human TM cells (Figure 5). The results showed that tunicamycin treatment significantly elevated the mRNA expressions of these fibrosis markers, and the changes were reduced with different concentrations of BRI, LAT, or OMD cotreatment significantly compared to the tunicamycin-treated group in human TM cells. Also, 1 and 10 μM LAT cotreatment showed significant suppression on α-SMA mRNA expressions compared to 0.1 μM LAT cotreatment group.

### 3.7. Comparison of BRI, LAT, and OMD on COL1A1, CTGF, Fibronectin, and α-SMA mRNA Expressions in Tunicamycin-Treated Human TM Cells

**Figure 6:**
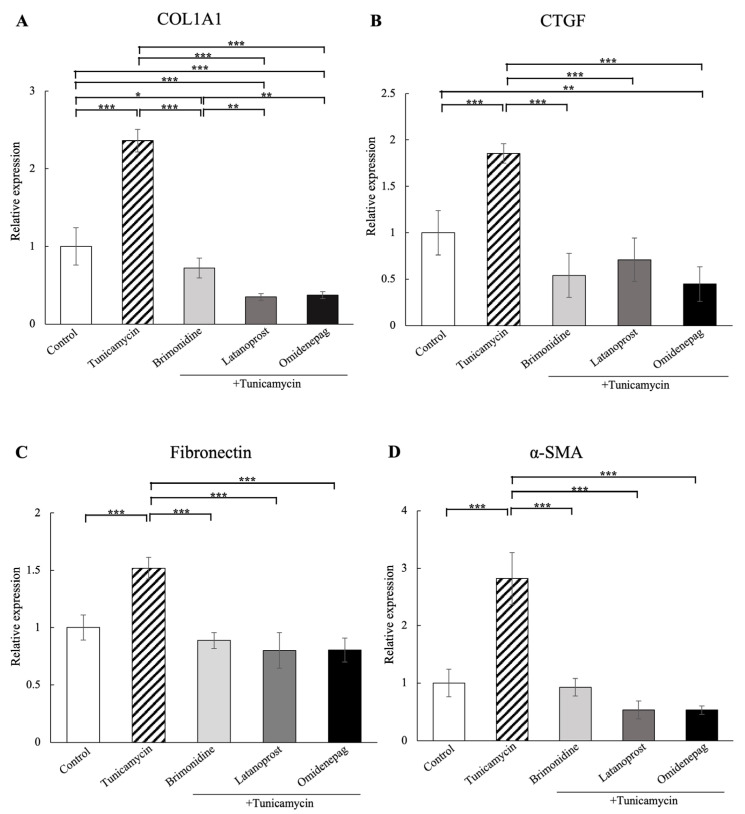
Real-time qPCR analysis of (**A**) COL1A1, (**B**) CTGF, (**C**) fibronectin, and (**D**) α-SMA mRNA expressions in human TM cells treated with 1 μM BRI, 0.1 μM LAT, or 0.1 μM OMD, with or without tunicamycin. Treatment with tunicamycin significantly elevated the mRNA expressions of COL1A1, CTGF, fibronectin, and α-SMA compared to the control group. In comparison with the tunicamycin-treated group, additional treatment of BRI, LAT, or OMD significantly decreased the upregulation in these fibrosis markers. In addition, cotreatment with LAT or OMD led to a significant reduction in COL1A1 mRNA expression compared to the BRI cotreatment group. GAPDH was used as an internal control for normalization. Data are presented as mean ± standard deviation. * *p* < 0.05, ** *p* < 0.01, *** *p* < 0.001.

We compared the effects of 1 μM BRI, 0.1 μM LAT, and 0.1 μM OMD on the mRNA expressions of fibrosis markers COL1A1, CTGF, fibronectin, and α-SMA in tunicamycin-treated human TM cells via real-time qPCR (Figure 6). The results indicated that the mRNA expressions of COL1A1, CTGF, fibronectin, and α-SMA were significantly raised with tunicamycin treatment, and the changes were lowered with BRI, LAT, or OMD cotreatment significantly compared to the tunicamycin-treated group. In addition, cotreatment with LAT or OMD significantly downregulated the COL1A1 mRNA expression compared to the BRI-cotreatment group in tunicamycin-treated human TM cells in vitro.

### 3.8. Effects of BRI, LAT, and OMD on COL1A1, Fibronectin, F-Actin, and α-SMA Protein Expressions in Tunicamycin-Treated Human TM Cells

**Figure 7:**
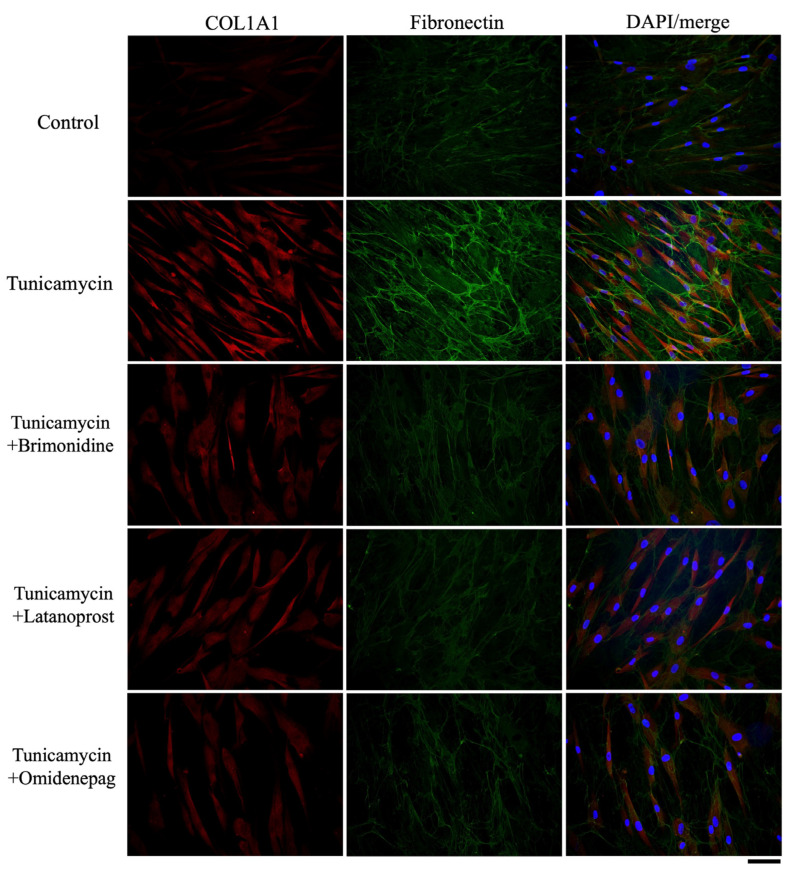
Immunocytochemistry of COL1A1 and fibronectin in human TM cells treated with BRI, LAT, or OMD, with or without tunicamycin. The left panels exhibit COL1A1 staining (red). The middle panels show fibronectin staining (green). The right panels present the merged image. Scale bar, 50 μm.

**Figure 8:**
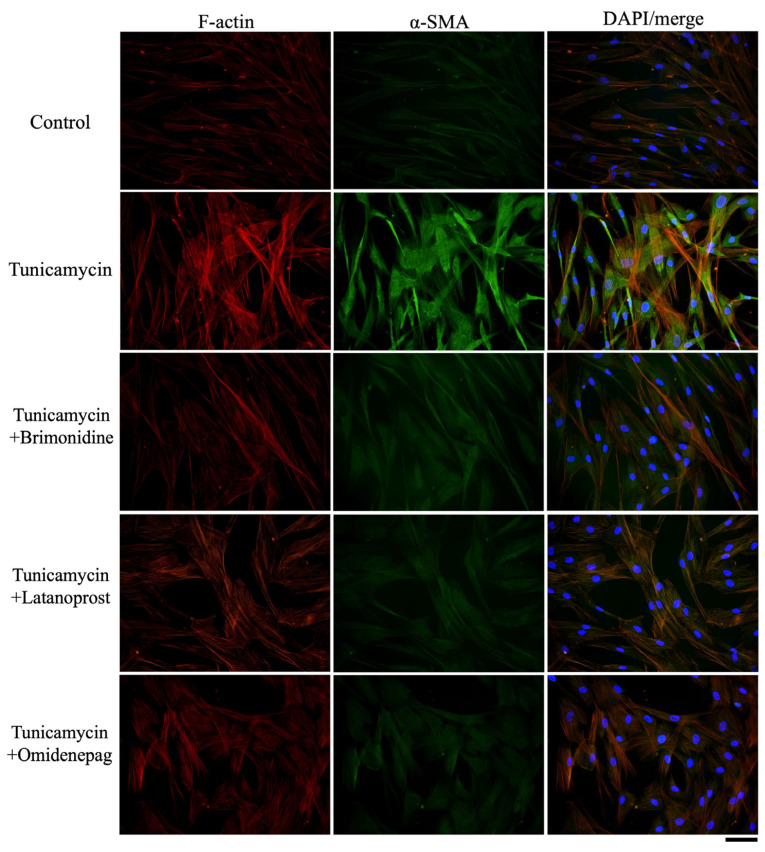
Immunocytochemistry of F-actin and α-SMA in human TM cells treated with BRI, LAT, or OMD, with or without tunicamycin. The left panels exhibit F-actin staining (red). The middle panels show α-SMA staining (green). The right panels present the merged image. Scale bar, 50 μm.

**Figure 9:**
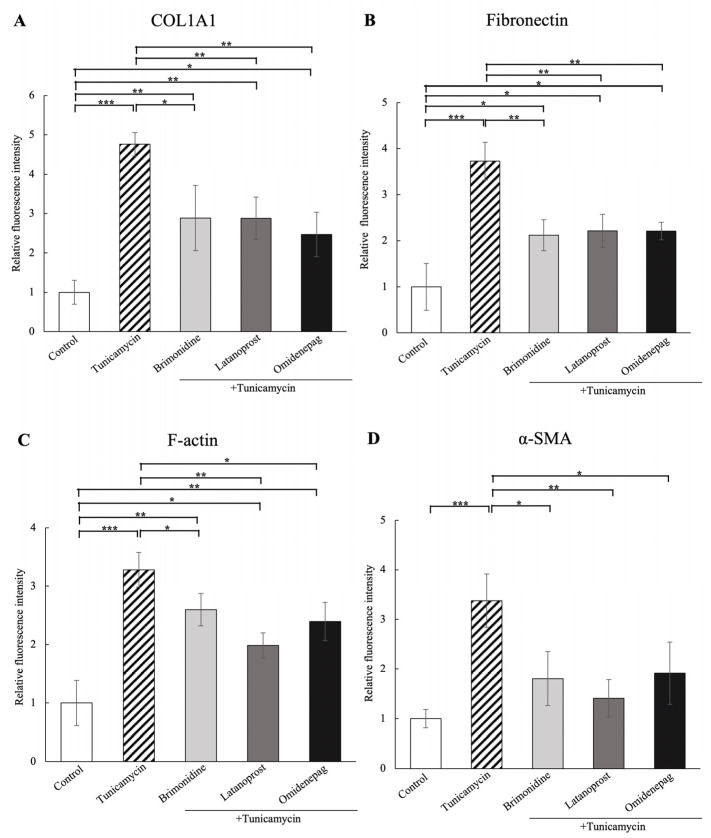
The quantitative results based on immunocytochemistry of (**A**) COL1A1, (**B**) fibronectin, (**C**) F-actin, and (**D**) α-SMA. The protein expressions of COL1A1, fibronectin, F-actin, and α-SMA were significantly upregulated with tunicamycin treatment, and the changes were downregulated with BRI, LAT, or OMD cotreatment significantly compared to the tunicamycin-treated group. Data are presented as mean ± standard deviation. * *p* < 0.05, ** *p* < 0.01, *** *p* < 0.001.

As shown in Figure 7, Figure 8 and Figure 9, immunocytochemistry was performed to investigate the effects of BRI, LAT, and OMD on the expressions of cytoskeletal proteins F-actin and fibrosis markers COL1A1, fibronectin, and α-SMA in tunicamycin-treated human TM cells in vitro. Tunicamycin treatment significantly elevated the expressions of these proteins. Cotreatment with BRI, LAT, or OMD significantly downregulated the F-actin, COL1A1, fibronectin, and α-SMA protein expressions compared to tunicamycin-treated human TM cells in vitro.

## 4. Discussion

In this study, we investigated the effects of tunicamycin on human TM cells, and further explored the effects of BRI, LAT, and OMD against ER stress and fibrosis of human TM cells treated with tunicamycin in vitro. Our results suggested that tunicamycin treatment significantly upregulated the mRNA expressions of the ER stress markers of CHOP, GRP78, and sXBP-1 and the fibrosis markers of COL1A1, CTGF, fibronectin, and α-SMA in human TM cells in vitro. Also, the protein expressions of CHOP, GRP78, sXBP-1, COL1A1, fibronectin, F-actin, and α-SMA were increased after treatment with tunicamycin significantly. With the additional cotreatment of BRI, LAT, or OMD, the damage to human TM cells was alleviated, and the changes to tunicamycin-induced human TM cells were minimized efficiently with each medication.

Studies have demonstrated that upregulated ER stress levels in TM tissues contribute to IOP elevation in POAG patients [13,16]. Under prolonged and severe ER stress, the accumulation of misfolded and unfolded proteins in the ER disrupts its homeostasis and leads to ER stress. In response to ER stress, CHOP is a mediator of cell apoptosis, ER chaperones GRP78 represents the overall level of ER stress, and sXBP-1 is reported to activate the apoptotic signaling pathway [12,39,40,41]. In our in vitro experiment, we found that a 1 μg/mL tunicamycin treatment significantly raised the ER stress markers of CHOP, GRP78, and sXBP-1 mRNA and protein expressions compared to the control in human TM cells. The results indicated that the presence of ER stress was extremely high and that cellular apoptosis was activated under tunicamycin-induced ER stress in human TM cells in vitro.

Subsequently, we investigated and compared the effects of glaucoma medications, BRI, LAT, and OMD on tunicamycin-induced ER stress in human TM cells in vitro. We observed that cotreatment with BRI, LAT, or OMD significantly downregulated the mRNA and protein levels of GRP78, which suggested the overall level of ER stress was reduced in tunicamycin-treated human TM cells. The upregulation of CHOP and sXBP-1 activate a proapoptotic signaling pathway under ER stress [41,42]. Reports demonstrated that CHOP mediates cellular dysfunction in ER stress, and CHOP deletion prevents IOP elevation in ER stress-induced hypertension mouse models [16,43]. Also, sXBP-1 inhibition was considered as a cytoprotective effect under ER stress [44]. In our study, cotreatment with LAT or OMD significantly reduced the mRNA and protein expressions of CHOP and sXBP-1 in tunicamycin-treated human TM cells. This finding may indicate that LAT or OMD treatment protects cells and alleviates cellular apoptosis in tunicamycin-treated human TM cells, which supports their anti-apoptotic effects in previous studies [22,29].

Excessive fibrosis accumulation is a major feature of TM tissues in POAG patients [45,46]. In addition, increased synthesis and deposition of fibrosis have been associated with ER stress and TM dysfunction [47,48]. Therefore, we also evaluated the effects of BRI, LAT, and OMD on the mRNA and protein expressions of cellular cytoskeleton and fibrosis markers in tunicamycin-treated human TM cells in vitro. As shown in Figure 5, Figure 6, Figure 7, Figure 8 and Figure 9, we observed significant upregulation in cellular cytoskeleton and fibrosis markers, including F-actin, COL1A1, CTGF, fibronectin, and α-SMA, in cells treated with tunicamycin. Our results support the previous finding that ER stress increases fibrogenic accumulation in human TM cells [49]. Cotreatment with BRI, LAT, or OMD significantly downregulated the upregulation of fibrosis markers compared to the tunicamycin-treated group, consistent with their reported antifibrotic effects [50,51,52]. It has been previously reported that BRI reduced transforming growth factor (TGF)-β2-induced fibrosis in human TM cells, which is in good accordance with our present result [53]. Furthermore, cotreatment with LAT or OMD significantly decreased COL1A1 mRNA expression compared to cotreatment with BRI, indicating superior downregulating effects against fibrosis in tunicamycin-treated human TM cells in vitro. Our result is in good accordance with the previous findings, including our previous report concerning the anti-fibrotic effects of OMD against TGF-β2-induced TM cell fibrosis [51]. Kalouche et al. [54] found that LAT decreases TM collagen accumulation but promotes cell contraction, possibly via activation of the p38 pathway, while another EP2 agonist of butaprost attenuates TM contraction and TGF-β2-induced collagen deposition by inhibiting the myofibroblast transition of TM cells. Although the remodeling effects of prostaglandin analogs on the uveoscleral outflow pathway have been investigated extensively and attributed to matrix metalloproteinase (MMP) activation, the absence of MMP modulation by LAT in the TM has been also reported [55,56]. Therefore, alternative mechanisms may be involved in the prostaglandin analog-mediated inhibition of extracellular matrix accumulation, potentially leading to increased AH drainage through the TM.

There are limitations in the present study. First, the concentration of medications may differ from clinical practice, although we used the reported anterior chamber concentration for each medication. Second, the specific conditions of the cultured medium may influence the observed protective effects shown in this study. Third, we evaluated only the short-term effects of these medications in vitro. The treatment of glaucoma needs a considerable amount of time, and a prolonged investigation would be required in a further study. Also, human TM cells express α2-adrenergic, FP, and EP2 receptors, regulating cAMP and Ca^2+^ levels [25,26,27,28,29,57,58,59]. The mechanism underlying the protective effects of these medications on ER stress-induced human TM cells will be needed for investigation in future experiments. We only evaluated and compared the effects of these three reagents on ER stress-induced human TM cells in vitro. A future study needs to focus on mechanisms using ER stress inhibitors to make the results more comprehensive. In this study, we investigated the cellular experiments alone; animal experiments using the ER stress model would need to be carried out to make the results more comprehensive in further studies.

To the best of our knowledge, this is the first study to evaluate and compare the effects of BRI, LAT, and OMD on tunicamycin-induced ER stress in human TM cells in vitro. A better understanding of the effects of these medications on ER stress may provide an effective and successful therapeutic outcome. Our findings suggest that cotreatment with BRI, LAT, or OMD protects human TM cells against ER stress and reduces fibrosis accumulation in vitro. It has been interesting to investigate whether tunicamycin affects human TM cell functions and how these drugs change tunicamycin-treated human TM functions. In this study, we evaluated the effects of BRI, LAT, and OMD in vitro with TM cells from healthy donors. An investigation into the effects of these three reagents on human TM cells from patients with poorly controlled glaucoma would be of great interest in a future study. The role of ER stress in POAG pathogenesis would be a potential new therapeutic target, and the molecular mechanisms of these medications against ER stress-induced human TM cells need to be comprehensively investigated in a further study.

## 5. Conclusions

In conclusion, our study suggests tunicamycin treatment induces ER stress and fibrosis in human TM cells in vitro. Cotreatment with BRI, LAT, or OMD alleviates tunicamycin-induced ER stress in human TM cells associated with POAG pathogenesis. The prevention of ER stress exposure to the TM may help to reduce the progression of POAG, which would be a therapeutic target for POAG.

## Data Availability

All data reported are provided in the text or in the figures.

## Supplementary Materials

The following supporting information can be downloaded at: https://www.mdpi.com/article/10.3390/biom15030389/s1, Figure S1: Human TM cell viability assay after treatment with different concentrations (0, 0.001, 0.01, 0.1, and 1 μg/mL) of tunicamycin for 24 h; Figure S2: Human TM cell viability assay after tunicamycin treatment at the different concentrations (0, 0.001, 0.01, 0.1, 1, 10, 30, and 50 μg/mL) for 24 h. File S1: Original western blots.
