# Supplementary material for: Effects of Brimonidine, Latanoprost, and Omidenepag on Tunicamycin-Induced Endoplasmic Reticulum Stress and Fibrosis in Human Trabecular Meshwork Cells
Source: Biomolecules. 2025 Mar 8;15(3):389. doi: 10.3390/biom15030389 (PMC11940208; doi:10.3390/biom15030389)
Supplement: Supplementary file 1 [file biomolecules-15-00389-s001.zip › biomolecules-3417255-supplementary figures.pdf]

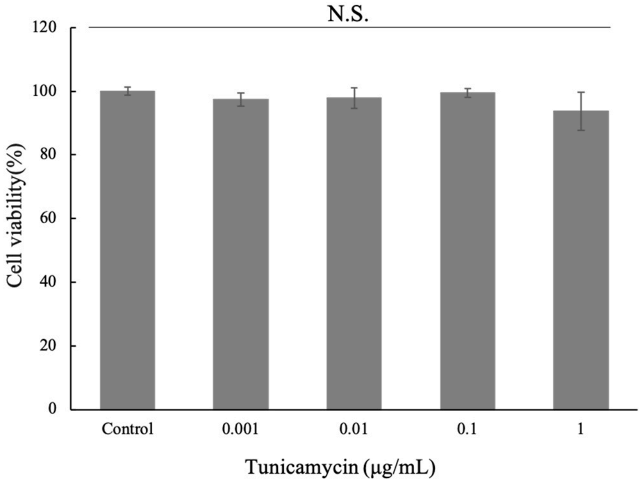

Supplementary Figure S1. Human TM cell viability assay after treatment with different concentrations (0, 0.001, 0.01, 0.1, and 1 μg/mL) of tunicamycin for 24h. The results showed that it did not induce significant changes in human TM cell viability compared to the control. Data are presented as the mean ± standard deviation.

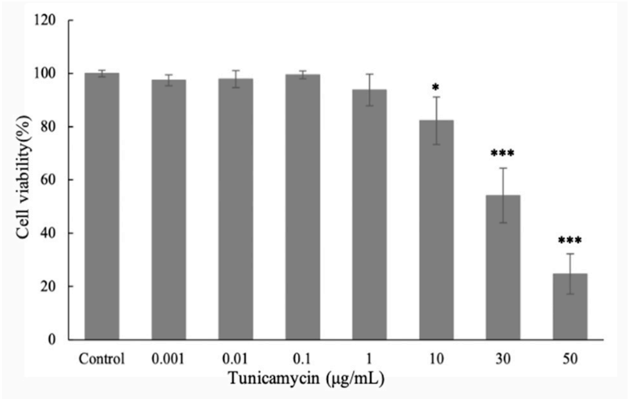

Supplementary Figure S2. Human TM cell viability assay after tunicamycin treatment at the different concentrations (0, 0.001, 0.01, 0.1, 1, 10, 30, and 50 μg/mL) for 24 h. The results showed that treated with 10, 30, and 50 μg/mL tunicamycin for 24 h significantly reduced cell viability compared to the control. Data are presented as the mean ± standard deviation.
