# Supplementary figures and images for: Effects of Brimonidine, Latanoprost, and Omidenepag on Tunicamycin-Induced Endoplasmic Reticulum Stress and Fibrosis in Human Trabecular Meshwork Cells
Source: Biomolecules. 2025 Mar 8;15(3):389. doi: 10.3390/biom15030389 (PMC11940208; doi:10.3390/biom15030389)

**Original gel images of Western blot for Figure 4.**

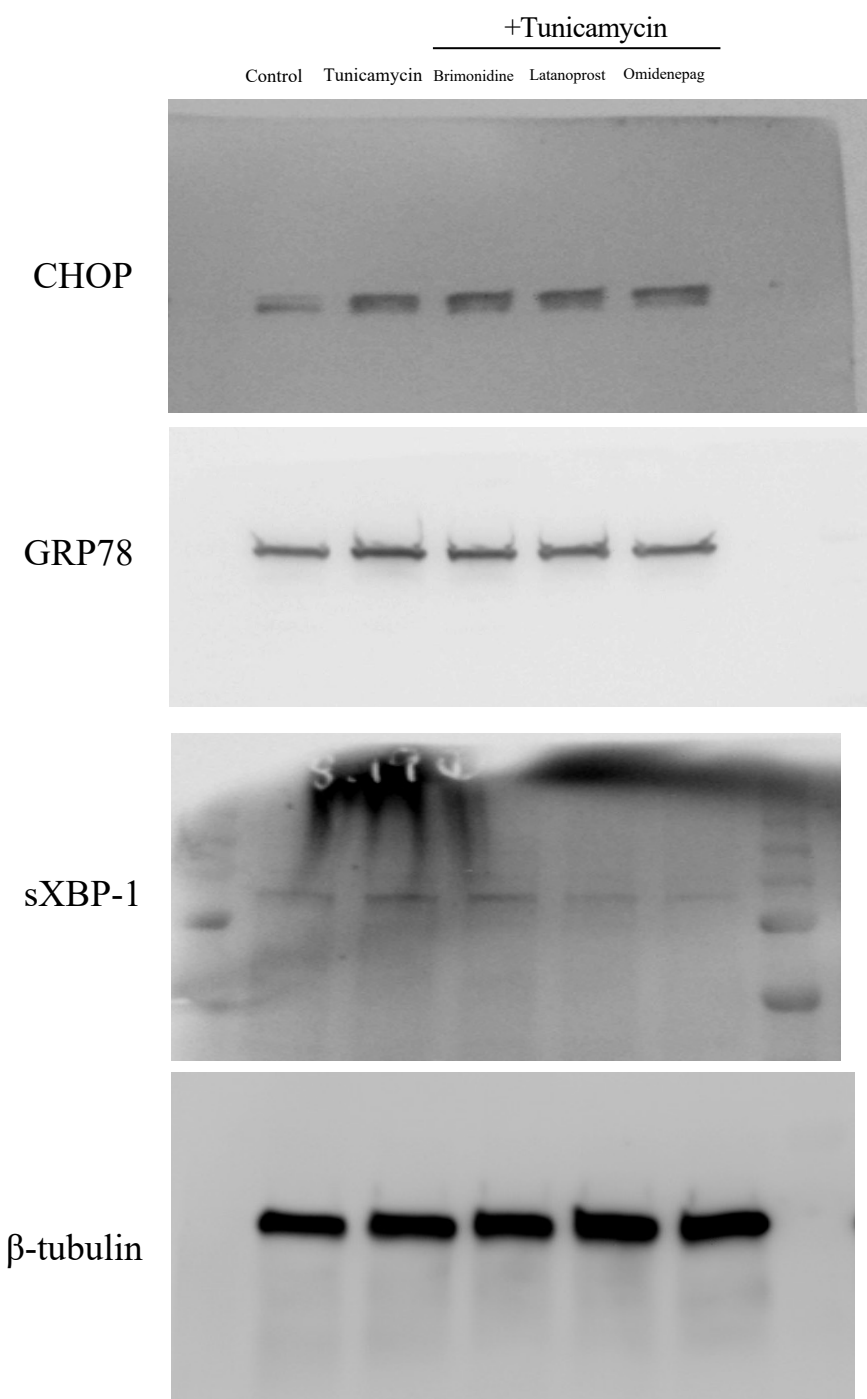

Supplement: Supplementary file 1 [file biomolecules-15-00389-s001.zip › biomolecules-3417255-File S1. original image.pdf]
